# Between-array normalization for 450K data

**DOI:** 10.3389/fgene.2015.00092

**Published:** 2015-03-10

**Authors:** Jonathan A. Heiss, Hermann Brenner

**Affiliations:** ^1^Division of Clinical Epidemiology and Aging Research, German Cancer Research Center (DKFZ)Heidelberg, Germany; ^2^German Cancer Consortium (DKTK)Heidelberg, Germany

**Keywords:** 450K, Infinium, normalization, quantile normalization, DNA methylation

## Abstract

The Illumina Infinium HumanMethylation450 BeadChip is frequently used in epigenetic research. Besides quantile normalization there is currently no standard method to normalize the data between arrays. We describe some properties of the data generated by this platform and present a normalization method based on local regression. We compare the performance of this method with other commonly used approaches in three benchmarks (correlation between 21 pairs of technical replicates, detection of differential methylation and correlation of methylation levels for smoking-associated CpG sites with smoking behavior of 655 participants of an epidemiological study). Results indicate that the proposed method improves reproducibility, whereas some commonly used methods can have adverse effects.

## 1. Introduction

DNA methylation (DNAm) is the modification of cytosine in CpG dinucleotides to 5-methylcytosine. DNAm is involved in cell differentiation, regulation of gene expression and development of cancer (Dawson and Kouzarides, 2012; Hackett and Surani, 2013). Methylation levels of specific CpG sites in blood samples have been found to reflect lifestyle factors (Lim and Song, 2012; Zhang et al., 2014) and have the potential to be used as biomarkers for early detection of cancer (Mikeska and Craig, 2014). In search of biomarkers, one is typically looking for differential methylation between two groups defined by a certain outcome. When measuring DNAm in blood samples, the sought-after changes can be small in magnitude. Therefore, obtaining precise measurements is of particular importance.

If DNAm is measured with high-density microarrays, there is often systematic bias between arrays due to a variety of variable experimental conditions such as concentrations of reagents or temperature, especially when the experiments are carried out in several batches (Lazar et al., 2013). Relevant biological signals may be masked by technical differences, also called batch effects and there are two fundamental ways to deal with them. One possibility is to consider batch effects in the statistical analysis, for instance by introducing a dummy variable for the batch in a linear model. However, batch effects may alter the data in complicated ways for which the statistical model in mind may not be adequate. It might therefore be preferable to remove these technical differences in a preprocessing step.

We have measured whole blood samples from participants of an epidemiological study with the Infinium HumanMethylation450 BeadChip (450K). In the following we describe a normalization method based on local regression, which we use to remove technical differences and to improve detection of relevant signals. We compare the performance of this method with other commonly used methods, which were previously reported to perform best (Marabita et al., 2013; Fortin et al., 2014; Wu et al., 2014), in three benchmarks using these data.

## 2. Methods

### 2.1. The chip

The 450K chip covers approximately 485,000 CpG sites out of the 28 million CpG sites in the human genome (Stirzaker et al., 2014). The design of this chip has been described in detail elsewhere (Bibikova et al., 2011). In the following we would like to point out some important properties of the data generated by this platform and introduce some notation.

- The chip uses single-base-extension of matched probe/target duplexes with dye-linked nucleotides and resultant fluorescence to measure abundance of unmethylated and methylated DNA molecules.
- The chip uses two different probe types, Infinium I and Infinium II (from now on abbreviated with *P*_1_ and *P*_2_). *P*_1_ is used for CpG dense regions like CpG islands, which are mostly unmethylated, *P*_2_ is used for CpG sparse regions, which are mostly methylated, so there are biological differences between the genomic loci covered by *P*_1_ and *P*_2_ (Eckhardt et al., 2006; Dedeurwaerder et al., 2011).
- For *P*_1_ the abundance of methylated and unmethylated DNA molecules of the same locus is measured in the same color channel, but with different beads/probes. By contrast, for *P*_2_ these molecules bind to the same beads, but are linked with different dyes: the unmethylated abundance is measured in the red color channel (Cy5) whereas the methylated abundance is measured in the green channel (Cy3). In the following, the intensity for methylated molecules from genome locus *i* and sample *z* is referred to by *M_iz_, U_iz_* denotes the intensity for unmethylated molecules. *P*_1_ consists of the subsets *P*_1*g*_ and *P*_1*r*_, the probes which are measured in the green or red color channel, respectively. Each can be further divided in the set of methylated or unmethylated signals (*P^M^*_1*g*_, *P^U^*_1*g*_, *P^M^*_1*r*_, *P^U^*_1*r*_, *P^M^*_2_, *P^U^*_2_).
- One cannot compare *M_iz_* and *M_i′z_* for *i* ≠ *i*′, due to the different binding affinities of the target molecules to the according probes. Additionally, one cannot compare *M_iz_* and *M_iz_*_′_ for *z* ≠ *z*′, because the concentrations of DNA and dyes vary from sample to sample. Both statements equally apply for the *U*-signals.
- Instead of comparing absolute signal intensities within and between samples one may calculate *m*-values *m_iz_* = ln $\frac{M_{iz}}{U_{iz}}$. For any given ratio of methylated to unmethylated molecules, *m_iz_* should not change with binding affinity or DNA concentration. Alternatively, one can compute β-values $\beta_{iz}=\frac{M_{iz}}{M_{iz}+U_{iz}}$, these fall in the range [0,1]. *m*- and β-values can be mutually transformed $\left(\beta_{iz}=\frac{{\text{e}}^{m_{iz}}}{{\text{e}}^{m_{iz}}+1};\;m_{iz}=\ln\frac{\beta_{iz}}{1-\beta_{iz}}\right)$.
- Nevertheless, *m_iz_* may still not be comparable between probes and between samples. Besides measurement errors, the *M* and *U* signals contain noise resulting from binding of off-target DNA molecules. In the pool of millions of species of DNA molecules, a lot of them share some non-negligible sequence similarity leading to cross-hybridization and changed equilibria (Horne et al., 2006). Approximately 6% of the probes have cross-reactive targets and these produce many false associations (Chen et al., 2013). If the noise for probe *i* is identically distributed between samples *z* and *z*′, then *m_iz_* and *m_iz_*_′_ are still comparable.
- For most probes there are replicates (multiple beads of the same type) on the chip. The software from the manufacturer reports summarized intensities.
- After amplification the abundance of target molecules exceeds the number of probe sequences on the chip.

### 2.2. Normalization

There are methods which focus on normalizing the *m*-values of *P*_1_ and *P*_2_ to each other within the arrays (Maksimovic et al., 2012; Teschendorff et al., 2013). This should be done carefully, as there are biological differences between the sites covered by the different probe designs. This step might not be necessary if subsequent analysis is done at the individual probe level. For instance, when computing *p*-values for single sites with the Wilcoxon-Mann-Whitney test for two groups of samples, precise but not accurate values are important. Also the *t*-test is invariant to scaling and shifting of the input values. For the detection of differential methylation at locus *i*, bias does not matter if it is the same for all samples. It is important to make *m*-values comparable across samples/arrays by removing the systematic biases between samples caused by batch effects.

A simple yet effective method is the quantile normalization (QN) (Bolstad et al., 2003; Marabita et al., 2013). However, it is not clear if this method is adequate for this kind of data. In the case of gene expression analysis the use of QN is justified by the assumption that only a tiny fraction of all genes is differentially expressed, therefore making the distribution of expression levels nearly equal, whereas the global methylation level may vary. In the following we present an approach which outperforms QN and is not based on such strong assumptions.

**Figure 1:**
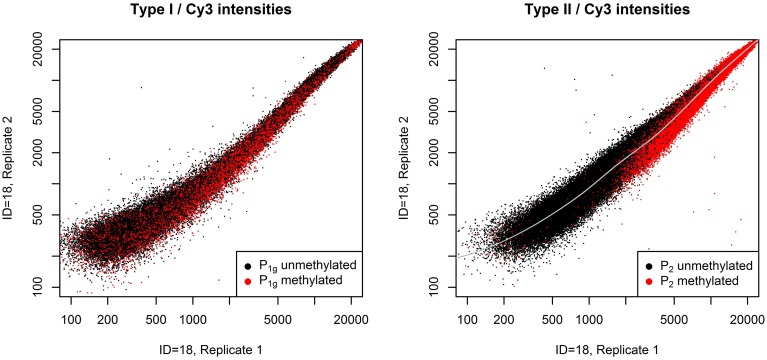
**Systematic bias between a pair of technical replicates**. Raw Cy3 intensities from a pair of technical replicates from dataset A (see section Benchmarks) for all probes in *P*_1*g*_ (left panel) or *P*_2_ (right panel). Probes with a *m*-value less than zero in the first replicate are plotted in black, otherwise in red. Probes in *P*_1_ show an intensity-dependent bias between the technical replicates, whereas for probes in *P*_2_ bias also depends on the methylation of the targets: unmethylated probes are mostly above the gray line, methylated probes below.

The normalization method we describe is based on the two plots shown in Figure 1. The left panel shows the raw Cy3 intensities of all probes in *P*_1*g*_ in a pair of technical replicates, the right panel shows the same for *P*_2_. Probes are colored according to their *m*-value in the first replicate. *P*_1_ exhibit an intensity-dependent bias between the two replicates, for *P*_2_ the bias also depends on the methylation of the targets. This violates one assumption of QN if applied to intensities, i.e., the assumption that the order of equally methylated targets does not change between samples.

First we try to correct the intensity-dependent bias by performing local regression on the intensity values. Second, we try to correct the methylation-dependent bias for *P*_2_ by performing a local regression on the *m*-values. Both times we use a set of “housekeeping CpG sites” and a virtual array made up of the medians over all samples as reference. The first step of the procedure was described previously (Wang et al., 2012), with the difference that we normalize both color channels separately and use only a subset of the probes to learn the loess curve. The exact steps are:

1. Adjust Cy3 intensities:
  - Take a set *E* of CpG sites which should be equally methylated in all samples *z* ∈ *Z*.
  - Compute for each probe *i* ∈ *E* ∩ *P*_1*g*_ a reference value *S*_i*_ = median(ln *S_iz_*), *z* ∈ *Z*. *S_iz_* can be an *M* or *U* signal. These values make up a virtual reference array which we use as baseline.
  - For each sample *z* ∈ *Z* perform a local regression with ln *S_iz_* as explanatory variable and ln *S_iz_*−ln S_i__*_ as dependent variable to learn the loess curve *f_g_*.
  - Compute the normalized intensities *S*′_*iz*_ = *S_iz_* · e^−*f_g_*(ln *S_iz_*)^. Normalize the Cy3 intensities of *P*_2_ using *f_g_* as well. Estimating a loess curve for *P*_2_ separately gives inferior performance, maybe because the two sources of bias (intensity-dependent, methylation-dependent) are entangled.
2. Adjust Cy5 intensities:
  - Repeat above steps for *P*_1*r*_ and the Cy5 intensities of *P*_2_.
3. Calculate *m*-values *m*′*_iz_* based on normalized intensities.
4. Adjust *m*-values of *P*_2_:
  - Take a set *E* of CpG sites which should be equally methylated in all samples *z* ∈ *Z*.
  - Compute for each probe *i* ∈ *E* ∩ *P*_2_ a reference value *m*_i*_ = median(*m*′_*iz*_), *z* ∈ *Z*.
  - For each sample *z* ∈ *Z* perform a local regression with *m*′*_iz_* as explanatory variable and *m*′*_iz_* − *m*_i*_ as dependent variable to learn the loess curve *f*_2_.
  - Compute the normalized *m*-values *m*″*_iz_* = *m*′*_iz_* − *f*_2_(*m*′*_iz_*).

Carrying out the above steps we get normalized *m*-values for all probes. For the set *E* CpG sites covering the exons of a list of housekeeping genes were used (Eisenberg and Levanon, 2013). This set is likely to contain some variable CpG sites, but local regression is robust against outliers. In total 843 probes for *P*_1*g*_, 1879 for *P*_1*r*_ and 5284 for *P*_2_ were used. A list of these probes is provided in the supplement. In contrast to the situation for cDNA microarrays, using housekeeping CpG sites for DNAm arrays does not suffer from the fact that the expression levels of these genes do not span the entire intensity range. There are some extreme values not contained in the interval of raw signal intensities or *m*-values of the reference array, but the numbers are low. For dataset A (see below) only 12,245 measurements (0.6‰) are affected.

### 2.3. Benchmarks

To assess the performance of different normalization methods we used 450K data from whole blood samples from participants of the ESTHER study (Breitling et al., 2011). In the ESTHER study patients aged between 50 and 74, who had a health check-up by their general practitioner, were recruited. Questionnaires for doctors and patients were used. These samples were combined to two datasets. Dataset A consists of 21 pairs of technical replicates distributed over 12 96-well plates, while no pair was allocated on the same plate. Dataset B consists of 655 samples (no replicates) together with age and self-reported smoking behavior (only data from current or never smokers were used) of the participants. The ESTHER study was approved by the ethics committee of the Medical Faculty of the University of Heidelberg and informed consent was obtained from all patients.

**Figure 2:**
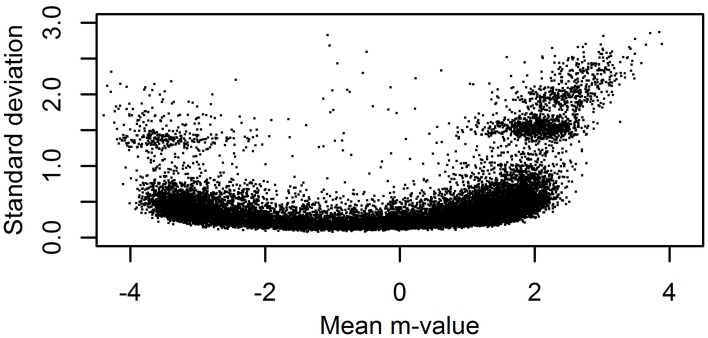
**Heteroscedasticity of *m*-values**. For a single 450K sample from the ESTHER study with unsummarized intensities the standard deviation and mean of single-bead *m*-values for probes in *P*_2_ with at least 20 beads on the chip were computed.

The performance of different normalization methods was evaluated in three benchmarks. The first benchmark tested if normalization increases the correlation between technical replicates of dataset A. As both measures of methylation are heteroscedastic (see Du et al., 2010 and Figure 2), we computed the correlation for *m*- and β-values. The former highlight the performance for mostly (un)methylated sites, the latter highlight the performance for sites with intermediate methylation. Non-CpG probes and probes located on the X and Y chromosome were excluded.

The second benchmark assessed which normalization method worked best for detecting differential methylation. Dataset A was splitted in half by splitting each pair of technical replicates up. For both halves samples were grouped by sex and probes ranked according to their *p*-values from a two-sample *t*-test (using *m*-values). We plotted the concordance between the two rankings from both halves by calculating the overlap percentage of the top *k* hits as done in Fortin et al. (2014). Again non-CpG probes and probes located on the X and Y chromosome were excluded in order to include QN in the benchmark. It does not matter for this benchmark if probes show differential methylation only due to cross-hybridization.

The third benchmark looked at known biomarkers for age and smoking. Spearman correlations of the methylation levels of three age-related CpG sites (Garagnani et al., 2012) with chronological age of the participants in dataset B were computed. Methylation of CpG site cg03636183 is strongly associated with current and long-term smoking exposure (Zhang et al., 2014). Spearman correlation of this biomarker with self-reported smoking behavior in dataset B was computed. Smoking exposure was assessed by three variables: **smoking status** with the categories never smoker (n = 469) or current smoker (n = 186), cumulative exposure (**packyears**) for current smokers, and the average number of cigarettes smoked per day (**numcig**) for current smokers. A detailed description of the variable definitions and the study population can be found in Zhang et al. (2014). For another 31 smoking-related sites [see Table 2 in Zeilinger et al. (2013)] Spearman correlations of methylation levels with **smoking status** were computed. Normalization should increase absolute values of the Spearman correlations.

We used.*idat* files for our analyses. All computations and statistical analyses were performed using R (R Core Team, 2014) and Bioconductor (Gentleman et al., 2004). R code for the first two benchmarks is provided online. As we normalized *P*_1_ and *P*_2_ differently, we also report the results for the first two benchmarks separately.

## 3. Results

We compared the normalization approach described above (LOESS) with the following: the standard method from the chip manufacturer without background correction (ILLU), the SWAN method (Maksimovic et al., 2012) and the FUNctional normalization (Fortin et al., 2014) as implemented in the *minfi* R package (Aryee et al., 2014). The BMIQ method (Teschendorff et al., 2013) with the R script version 1.3 downloaded from code.google.com/p/bmiq/We. QN applied to intensities of *P*_1*g*_, *P*_1*r*_, *P^M^*_2_ and *P^U^*_2_ separately (QN1); this way each combination of color channels and probe types is normalized separately. QN applied to raw *m*-values of *P*_1*g*_, *P*_1*r*_ and *P*_2_ separately (QN2). For QN1 and QN2 only autosomal probes were normalized, as dataset A includes samples from both sexes.

**Figure 3:**
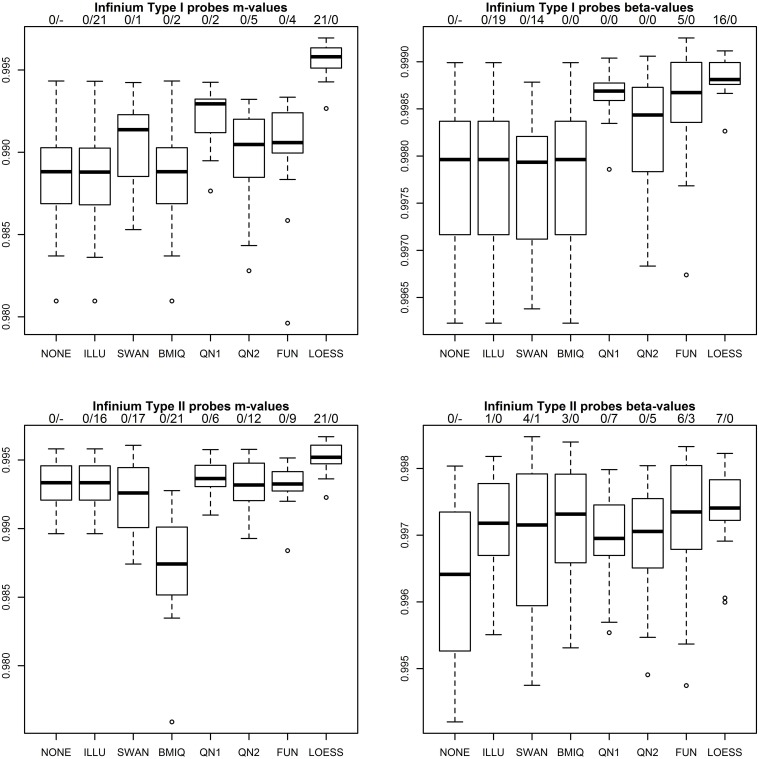
**Correlation of *m*-values and β-values between technical replicates after various normalization methods for *P*_1_ (top row) and *P*_2_ (bottom row)**. Methods used include raw values (NONE), the standard method in the GenomeStudio software from the chip manufacturer (ILLU), the SWAN method (Maksimovic et al., 2012), the BMIQ method (Teschendorff et al., 2013), quantile normalization applied to intensities (QN1) and to *m*-values (QN2), FUNctional normalization (Fortin et al., 2014) and the method described in this paper (LOESS). Two numbers on top of each boxplot and normalization method indicate how often the method achieved the highest correlation for a pair of technical replicates and how often the correlation declined compared to the raw values.

Figure 3 shows the results of the first benchmark for the four combinations of probe type (*P*_1_, *P*_2_) and methylation measure (*m*, β). Two numbers on top of each boxplot and normalization method indicate how often the method achieved the highest correlation for a pair of technical replicates and how often the correlation declined compared to the raw values. As expected for ILLU and (*P*_1_, *m*),(*P*_1_, β),(*P*_2_, *m*) the correlations are virtually unchanged, as this method mainly scales intensities. Only for (*P*_2_, β) it increased correlations. SWAN increased correlations for (*P*_1_, *m*) and (*P*_2_, β), but had adverse effects for (*P*_1_, β) and (*P*_2_, *m*). As expected for BMIQ correlations for (*P*_1_, *m*) and (*P*_1_, β) were virtually unchanged, but it reduced the correlations for (*P*_2_, *m*) for all 21 pairs of technical replicates. QN1 performed well for (*P*_1_, *m*) and (*P*_1_, β), but produced mixed results for (*P*_2_, *m*),(*P*_2_, β). QN2 was only favorable for (*P*_1_, β). FUN performed well for (*P*_1_, β), but produced mixed results for (*P*_1_, *m*),(*P*_2_, β) and had adverse effects for (*P*_2_, *m*). LOESS achieved the highest correlation for all 21 pairs of technical replicates for (*P*_1_, *m*),(*P*_2_, *m*) and had a clear lead for (*P*_1_, β),(*P*_2_, β). It was the only method that improved correlation in every single case.

**Figure 4:**
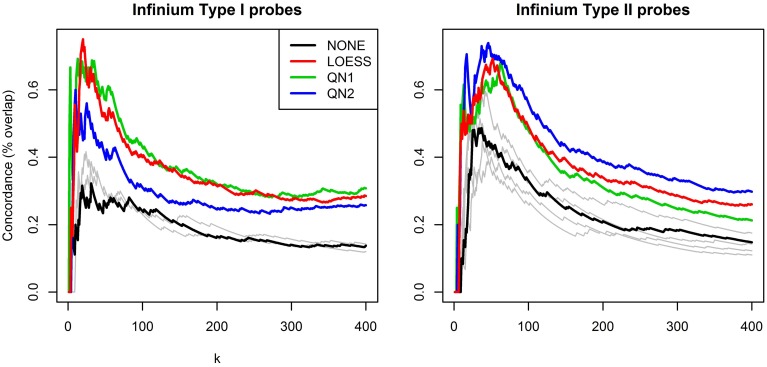
**Concordance between rankings for differential methylation**. Technical replicates from dataset A were splitted to discovery and validation set and subsequently grouped by sex. Probes were ranked according to *p*-values from a two-sample *t*-test. The plots show overlap percentage between the two rankings for the top *k* hits for *P*_1_ and *P*_2_ separately. For clarity, only the best performing methods are highlighted, the rest are plotted in gray.

Figure 4 shows the results of the second benchmark. For the sake of clarity only the lines for NONE and the three best performing methods, LOESS, QN1 and QN2, are highlighted, and *k* is limited to [1,400], as for higher values the order of the lines did not change. QN1 and LOESS were on par for *P*_1_, followed by QN2. QN2 performed best for *P*_2_, followed by LOESS and QN1. Some methods had again adverse effects.

Table 1 shows the results of the third benchmark for the age-related biomarkers and cg03636183. In all cases LOESS improved the Spearman correlation (for cg06639320 and age about 0.16) and it achieved the highest absolute value of all normalization methods in 4 out of 6 cases. The results for the other 31 smoking-related sites are provided in the supplement. In summary, for 26 out of 31 CpG sites LOESS improved the correlation (ILLU 26, SWAN 25, BMIQ 25, QN1 24, QN2 25, FUN 27) and it achieved the highest absolute value for 12 CpG sites (NONE 1, ILLU 2, SWAN 3, BMIQ 1, QN1 3, QN2 9, FUN 0). 28 of the 31 sites are in *P*_2_.

**Table 1:** **Results of the third benchmark**.

**Trait**	**Biomarker**	**NONE**	**ILLU**	**SWAN**	**BMIQ**	**QN1**	**QN2**	**FUN**	**LOESS**
Smoking status	cg03636183	−0.665	−0.674	−0.678	−0.672	−0.674	−0.676	−0.671	−0.679
Packyears	cg03636183	−0.324	−0.355	−0.353	−0.335	−0.351	−0.328	−0.338	−0.346
Numcig	cg03636183	−0.281	−0.320	−0.328	−0.305	−0.331	−0.328	−0.303	−0.345
Age	cg16867657	0.648	0.648	0.605	0.648	0.672	0.648	0.654	0.682
Age	cg06639320	0.383	0.461	0.462	0.441	0.484	0.457	0.446	0.490
Age	cg16419235	0.441	0.441	0.431	0.441	0.468	0.441	0.441	0.458

*Spearman correlations of age or smoking behavior with methylation levels of known biomarkers*.

## 4. Discussion

We show that the normalization method described here reduces the differences between technical replicates more effectively than QN and improves detection of differential methylation. For known biomarkers the associations of methylation levels with the respective traits become more evident. At the same time our method does not rely on such strong assumptions like QN, and probes located on the sex chromosomes can be normalized as well. Only a small set of sites (~1.6% of all probes) equally methylated across all samples is required. This makes LOESS especially useful when QN cannot be applied, for example in case of global methylation changes between samples.

For the detection of differential methylation one should be careful when using within-array normalization, as in our analysis these methods had often adverse effects. Most importantly we argue that, although different probes are not comparable even if they are of the same probe type, this does not affect detection of differential methylation in a univariate screening. If there is need for accurate quantification of methylation levels, other experimental methods should be considered. We also show that even when the assumption of equal distribution of methylation levels between samples is valid, absolute intensity values of *P*_2_ are still not comparable. The following argument might justify the use of QN1, at least for *P*_1_. Suppose there are two samples with an identical epigenome. If one changes an arbitrary number of completely unmethylated CpG sites (of type *P*_1_) to completely methylated or vice versa, the distribution of intensity values stays the same (high *U* signal and low *M* signal → low *U* and high *M* signal in th same color channel with the same affinity).

Most of the time the separation between low and high *m*-values is not that clear as in Figure 1. We do not know the cause of this pattern. It may be due to a combination of competitive effects (remember that DNA is in excess) and different binding affinities for unmethylated and methylated targets (but probes in Figure 1 do not cluster according to the number of underlying CpG sites).

Of course not all batch effects can be removed by our method. In particular we consider bias connected to signal intensities and methylation levels. However, there might be other sources of bias, like bias connected to the sequence of a probe. A good study design is still crucial.

Our results for SWAN are not in conflict with the evidence presented by Maksimovic et al. (2012), as they only looked at distributions of methylation levels, which have no significance for the conclusions they have drawn, also noted by Pidsley et al. (2013). An extensive evaluation of existing normalization methods can be found in Marabita et al. (2013) and Wu et al. (2014). The authors also tested SWAN and show that this method can reduce the correlation between technical replicates. The results for the methods ILLU and QN1 are not comparable with these papers, since we performed ILLU without background correction, because it was unfavorable, and normalized *P*_1*g*_, *P*_1*r*_, *P^U^*_2_ and *P^M^*_2_ separately for QN1. Marabita et al. recommend a combination of QN and BMIQ.

One strength of our work is the high number of samples used in the benchmarks, 21 pairs of technical replicates and 655 blood samples from a well-described study population, collected and handled in a consistent way. There are however some limitations of this work. We used correlation as a measure of similarity of replicates in the first benchmark. A distance measure, which accounts for the heteroscedasticity of the data, would be more adequate. Another issue is the set of housekeeping CpG sites. We used probes covering exons of genes showing constant expression levels across a wide range of tissues (Eisenberg and Levanon, 2013). Identifying probes with constant methylation directly would be a better approach, but this would require a dataset of samples from different tissues measured on the same 450K plate, in order to minimize batch effects. Although the selected CpG sites should work for a wide range of tissues, we did not test this. Certainly this set needs to be refined as it will contain many sites which are not invariant. In the current implementation, measurements outside the range of values in the reference set are discarded, extrapolating would reduce the number of missing values. We also did not test how this normalization method operates with different parameter settings for the LOESS function in order to avoid overfitting. There is need for further investigations of these issues.

### Conflict of interest statement

The authors declare that the research was conducted in the absence of any commercial or financial relationships that could be construed as a potential conflict of interest.
